# Pectoralis Minor Syndrome: Case Presentation and Review of the Literature

**DOI:** 10.1155/2016/8456064

**Published:** 2016-06-26

**Authors:** Mohammed Abdallah, Mohammad Rachad Wehbe, Elias Elias, Muhammad Aghiad Kutoubi, Roger Sfeir

**Affiliations:** ^1^Division of General Surgery, Department of Surgery, American University of Beirut Medical Center, P.O. Box 11-0236, Riad El-Solh, Beirut 1107 2020, Lebanon; ^2^Division of Surgical Oncology, Department of Surgery, King Hussein Cancer Center, Amman, Jordan; ^3^Division of Neurosurgery, Department of Surgery, American University of Beirut Medical Center, P.O. Box 11-0236, Riad El-Solh, Beirut 1107 2020, Lebanon; ^4^Department of Diagnostic Radiology, American University of Beirut Medical Center, Beirut, Lebanon

## Abstract

We present a case of a healthy young female with axillary vein compression caused by the pectoralis minor muscle. Diagnosis was made by clinical findings and dynamic venography. After pectoralis minor tenotomy, the patient had total resolution of her symptoms. Compression of the axillary vein by the pectoralis minor is a rare entity that needs a careful exam and imaging to reach its diagnosis and establish the appropriate treatment.

## 1. Introduction

Thoracic outlet syndrome is characterized by nonthrombotic venous obstruction caused by compression of the upper extremity's neurovascular bundle in the scalene triangle bordered by the clavicle, first rib, and scalene muscles above the clavicle [1]. However a mimicking infrequent entity, labeled the pectoralis minor syndrome (PMS), is described in the literature. It is characterized by axillary vein obstruction below the clavicle, provoked by compression from the pectoralis minor muscle [2]. Clinical presentation is portrayed as pain, weakness, cyanosis, numbness, paresthesia, and swelling of the upper extremity [3]. It is rarely on the physician's differential diagnosis and mostly neglected as an etiology and is considered as a subset of thoracic outlet syndrome. We present a case of nonthrombotic axillary vein obstruction caused by the pectoralis minor (PM), highlighting the role of venography and treatment modality by pectoralis minor tenotomy (PMT).

## 2. Case Presentation

A 29-year-old healthy female presented to our hospital with complaints of recurrent swelling and pain of the right upper extremity. The incident started eight years prior to presentation, happening once every three to five months but increasing in frequency over the previous three months. Episodes were associated with tingling and numbness sensation of the hand along with bluish discoloration of the fingers. Swelling was present at rest and aggravated upon exercise and/or elevation of the arm. She recalled history of right upper extremity trauma 12 years before. She had multiple emergency department visits, during which she endured multiple lab tests and imaging studies, including venous duplex of the upper extremity revealing no evidence of either deep or superficial vein thrombosis. Other investigations done such as chest and cervical spine X-rays showed no cervical rib or cervical pathology. Venography and MR venography showed no evidence of thoracic outlet syndrome. EEG-EMG of the upper extremity showed normal motor and sensory conduction, with no electrophysiological evidence of cervical root disease, brachial plexus lesion, thoracic outlet syndrome, or focal nerve entrapment in the right upper extremity.

On physical exam, she had swelling in the right upper extremity; no point tenderness over supra- or infraclavicular regions was noticed, with palpable brachial, radial, and ulnar pulses in resting position and in Adson's maneuver. Lastly, elevated arm stress test was negative.

**Figure 1:**
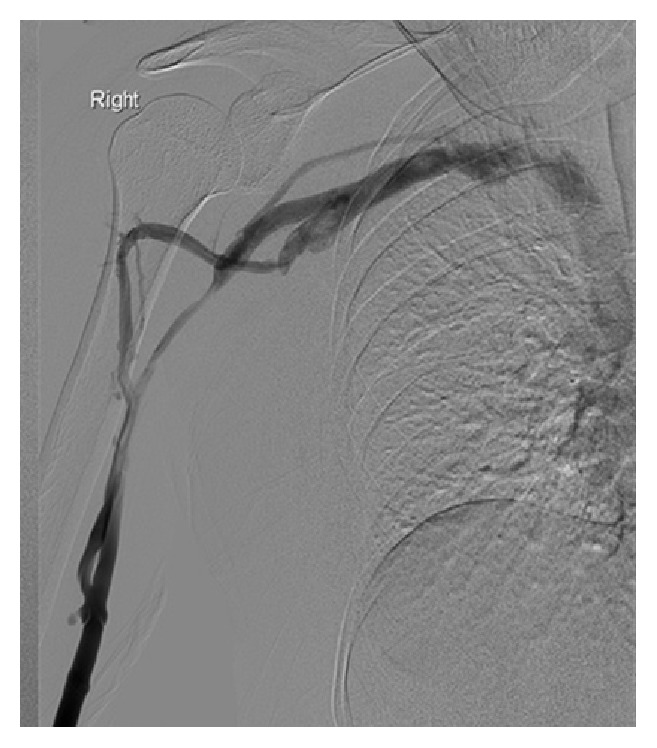
(Neutral position pic.) Resting position with tourniquet: pectoralis muscle stretched by fixing the breast medially: venography was performed by injecting 20 mL of dilute contrast agent through the catheter, showing occlusion of the medial basilic vein and narrowing of the lateral.

**Figure 2:**
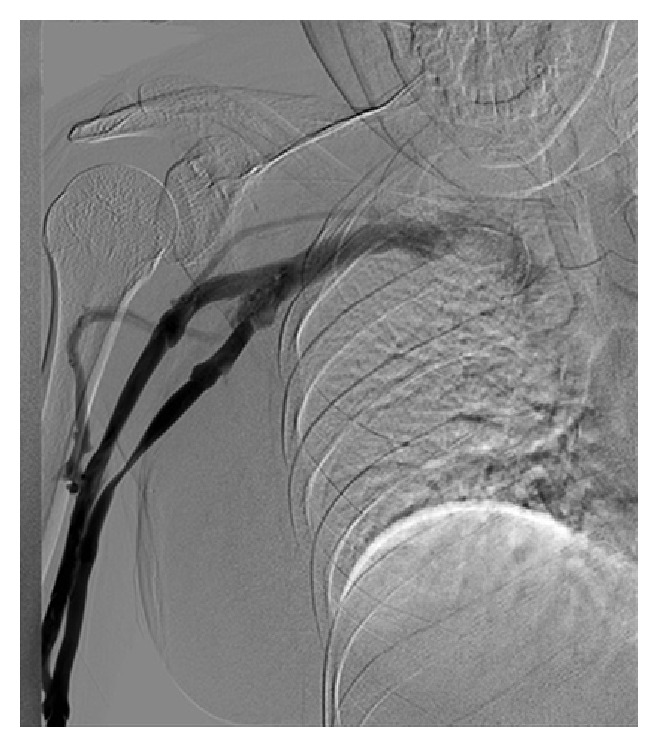
Arm pulling. Dynamic venography with the arm pulled inferiorly and then released: venography was performed by injecting 40 mL of dilute contrast agent through the catheter, showing obstruction of the basilic/axillary veins and reflux and filling of the small cephalic vein providing collateral circulation filling the subclavian vein. The occlusion of the axillary vein was completely resolved upon releasing the tension. Findings of both pictures (Figures 1 and 2) in keeping with pectoralis minor syndrome on the right side. No venous compression at the costoclavicular space or the thoracic inlet.

Repeat conduction and upper duplex studies in our institution were normal. However, when performing venography using a catheter inserted in the basilic vein (Figures 1 and 2), it showed no significant abnormality in resting position nor in Adson's maneuver with the arm elevation and 180-degree abduction. But when the PM was stretched medially with the arm pulled inferiorly before being released, an obstruction of the basilic/axillary veins was noticed, associated with reflux and filling of the small cephalic vein, providing collateral circulation, filling the subclavian vein, which completely resolved upon releasing the tension. Contralateral side venography with the same maneuver was normal, with no evidence of axillary vein thrombosis.

## 3. Surgical Procedure

Under general anaesthesia, the patient underwent right pectoralis minor tenotomy (PMT) via transaxillary approach. A 5 cm incision was carried out, started 1 cm above the bottom of the hairline. The pectoralis major muscle was separated from the pectoralis minor and retracted anteriorly. The PM muscle was found hypertrophied; it was transected proximal to its insertion on the coracoid process avoiding injury to the pectoral nerve. No other pathology was found to compress the axillary vein. The patient tolerated the procedure well and was discharged the next day. At follow-up six months after the operation, she was doing well, free of symptoms, leading a normal daily life activity.

## 4. Discussion

Causes of PMS vary. It could be due to traumatic accidents, sports injuries caused by repetitive movements, or weight lifting or as a result of idiopathic or spontaneous occurrence [3]. Most common presentations are upper extremity paresthesia and pain in the subclavicular anterior chest wall and axilla. Arm cyanosis and swelling are other accompanying variables [2]. Unlike Neurogenic Thoracic Outlet Syndrome (NTOS) which is a disorder produced by compression of the components of the brachial plexus nerves, PMS is less likely to be associated with cervical pain or headache [4]. In brachial plexus compression, the simultaneous finding of both thoracic outlet and pectoralis minor areas involvement is common in the same patient, which is not the same for arteriovenous compression [5].

Hidden under the PM are the axillary artery and vein along brachial plexus nerves which are more likely to endure extrinsic compression due to proximity of PM tendon. It is important to distinguish the obstruction site by differentiating between axillary and subclavian vein in order to plan for the appropriate treatment measures.

Diagnostic modalities include electrophysiological studies such as EMG, which is usually normal in PMS but should be ordered to rule out neurological conditions. Although duplex scan is sensitive to detect venous thrombosis, it is unlikely to identify subtle degree of compression [6]. Instead, dynamic venography is considered the most helpful diagnostic tool in PMS [2].

Treatment procedures vary from PM stretching to PM block [3] which were avoided in our case since the patient denied any pain or paresthesia over the pectoralis minor muscle and its tendon. When such conservative measures fail, PMT, a low risk surgical procedure, becomes the modality of treatment. It could be approached via two different techniques. The infraclavicular method is conducted by incising 3 cm below the clavicular center while the transaxillary approach is carried by cutting the skin 1 cm above the bottom of the axillary hairline [7]. This decreases the injury risk to the second intercostal-brachial cutaneous nerve [8]. Recovery time postoperatively is often few days, with recommendation of refraining from using the arm for activities above the level of the shoulder for 2-3 months in order to permit the adherence of the divided PM muscle to the chest wall [3]. Success rate is higher when the PMS is diagnosed solely compared to an association with other entities such as NTOS [9].

## 5. Conclusion

Axillary venous compression by the pectoralis minor is a rare entity that presents with symptoms similar to subclavian vein obstruction. Treatment is surgical by PMT, a low risk procedure that projects good outcome.
